# Polysomnography harmonization schemes: A multi-institutional sleep research resource

**DOI:** 10.1371/journal.pone.0361116

**Published:** 2026-11-05

**Authors:** Yujin Han, Gayeon Ryu, Yeongdon Kim, Hangsik Shin

**Affiliations:** 1 Department of Medical Informatics and Statistics, Brain Korea 21 Project, Asan Medical Center, University of Ulsan College of Medicine, Seoul, Republic of Korea; 2 Department of Medical Science, Graduate School, University of Ulsan, Seoul, Republic of Korea; 3 Institute of Digital Healthcare, University of Ulsan College of Medicine, Seoul, Republic of Korea; Politecnico di Torino, ITALY

## Abstract

Sleep disorders are associated with cardiovascular diseases and cognitive decline worldwide, highlighting the need for effective therapeutic strategies. Polysomnography (PSG) is the gold standard diagnostic tool for sleep disorders. However, long examination times, high costs, specialized biosignal equipment and controlled settings requirements hinder large-scale data collection. As artificial intelligence applications in sleep research expand, structural heterogeneity in PSG data across institutions complicates multi-center integration, increases preprocessing burden, and limits reproducible large-scale algorithm validation. We investigated biosignals, sleep parameters, and demographic features from eight publicly available PSG datasets. A set of schemas for PSG harmonization is proposed to formalize unified structural specifications aligned with established clinical guidelines. These schemas systematically integrate heterogeneous biosignals, derived sleep parameters, and demographic features to achieve consistent attribute selection and uniform preprocessing across datasets. When applied to construct an integrated dataset, the schemas demonstrated stable demographic and clinical distributions through repeated sampling. By addressing schema-level interoperability, the proposed framework facilitates consistent algorithm evaluation and comparison and provides a structured reference for reproducible, large-scale sleep research.

## Introduction

Polysomnography (PSG) is the gold standard diagnostic method for sleep disorders. PSG simultaneously records multiple physiological signals during sleep, including electroencephalography (EEG), electrooculography (EOG), electromyography (EMG), electrocardiography (ECG), respiratory airflow, respiratory effort, and oxygen saturation [1]. Because PSG requires overnight monitoring using multiple sensors in a specialized clinical environment, large-scale data acquisition becomes resource-intensive, time-consuming, and costly. Nevertheless, the increasing availability of publicly accessible PSG repositories has enabled large-scale sleep research and has accelerated the development of artificial intelligence (AI)-based approaches for automated sleep analysis.

The National Sleep Research Resource (NSRR; https://sleepdata.org/) provides access to 28 publicly available PSG datasets based on large-scale epidemiological cohorts [2]. Since its establishment, NSRR has released over 30,000 PSG recordings in conjunction with corresponding clinical data. Among these, the Sleep Heart Health Study (SHHS), Multi-Ethnic Study of Atherosclerosis (MESA), and Osteoporotic Fractures in Men Study (MrOS) have been widely used in studies of automated sleep apnea detection, sleep stage classification, and other sleep disorders [3–5]. Similarly, PhysioNet (https://physionet.org/) provides publicly accessible PSG datasets collected from multiple institutions and clinical settings [6].

However, structural heterogeneity remains a critical barrier to integrative analysis. Differences in acquisition hardware, electrode placement, signal naming conventions, sampling frequencies, annotation formats, and derived parameter definitions have led to inconsistencies across datasets. Although repositories such as NSRR provide harmonized terminology and data dictionaries for selected clinical variables, the original biosignal configurations of each dataset are largely preserved. Even within NSRR, each dataset retains its original montage and sampling specifications, requiring researchers to manually interpret documentation files before these datasets can be systematically utilized for sleep research. Such multi-center heterogeneity complicates reproducible cross-dataset analysis and limits scalability in combined AI modeling efforts [2,7,8].

An effort to address PSG harmonization is the Common Sleep Data Pipeline (CSDP), which proposed a standardized preprocessing framework for harmonizing input biosignals across multiple sleep datasets [8]. CSDP standardizes signal-level processing steps such as channel mapping, filtering, scaling, and resampling to support consistent model development across heterogeneous PSG datasets. Although this approach reduces variability at the signal-processing level, it does not define a comprehensive structural schema integrating raw biosignals, sleep parameters, and demographic features into a unified conceptual specification. Consequently, existing repositories have improved metadata accessibility and preprocessing pipelines and standardized signal-level workflows, although a formalized schema-level framework for integrating heterogeneous PSG datasets remains limited.

The primary objective of this study was to develop and validate an extensible harmonization schema for heterogeneous multi-center PSG datasets aligned with the American Academy of Sleep Medicine (AASM) manual. Unlike approaches limited to signal preprocessing or task-specific parameter selection, we systematically analyzed and formalized the names, definitions, units, and structural representations of biosignals, sleep events, and demographic features across selected publicly available datasets. By comparatively examining structural differences among datasets, we constructed a unified schema that provides a consistent conceptual and structural specification for PSG harmonization. To the best of our knowledge, no prior study has formalized an extensible harmonization framework across multiple publicly available PSG datasets by integrating biosignals, sleep parameters, and demographic features within a unified structural specification grounded in established clinical guidelines.

The proposed schema was subsequently applied to publicly available PSG datasets to construct a consistently structured integrated dataset. To evaluate the feasibility of constructing a consistently structured test set, repeated sampling was performed, and the reproducibility of the demographic and clinical features of the test sets was assessed. By addressing schema-level interoperability, this study aimed to establish a data-infrastructural foundation for reproducible multi-cohort sleep research and AI model benchmarking across heterogeneous PSG datasets.

## Materials and methods

### Source datasets for schema design

The schemas for PSG harmonization were developed based on the following eight publicly available PSG datasets from the NSRR: Apnea, Bariatric Surgery, and CPAP Study (ABC); Apnea Positive Pressure Long-term Efficacy Study (APPLE); Cleveland Family Study (CFS); MESA; MrOS; SHHS; Study of Osteoporotic Fractures (SOF); and Wisconsin Sleep Cohort (WSC) [2,9–16]. These datasets were collected in distinct clinical contexts and involve heterogeneous participant populations. Thus, these source datasets were applied to design the schemas for PSG harmonization. The selected datasets differ in demographic composition depending on the study purpose of each cohort. Information on the study purpose of each dataset, PSG acquisition type, sample size, sex distribution, and age range was obtained from the official NSRR documentation (S1 Table). The data were accessed for research purposes on April 22, 2024, except for the MrOS dataset, which was accessed on April 28, 2025. The authors had no access to personally identifiable information at any time, as all datasets obtained from the NSRR are fully de-identified.

The NSRR PSG datasets are provided in three formats: European data format (EDF), comma-separated values (CSVs), and extensible markup language (XML). The EDF files contain raw biosignals, including EEG, ECG, EOG, chin or submental EMG, leg EMG, airflow, respiratory effort, oxygen saturation, and body position.

The XML files include annotations scored manually by sleep technicians. Sleep stages are labeled on a 30 s epoch basis, whereas scored events, including respiratory events such as sleep apneas and hypopneas, arousals, limb movements, and cardiac events, are recorded separately as event-level annotations with event type, onset time, and duration. The scoring definitions and parameters for these annotations were reviewed based on the cohort-specific NSRR documentation and scoring manuals, and the original annotations were used without re-scoring or modification.

The CSV files contain demographic information and quantitatively derived sleep parameters such as total recording time (TRT) and sleep efficiency. To construct the schemas for PSG harmonization, we investigated all the signal and parameter attributes listed in the EDF files (containing biosignals) and CSV files (containing sleep parameter files of the selected datasets) and compiled a unified list based on the AASM Manual for the Scoring of Sleep and Associated Events [17].

### Construction of the schemas for PSG harmonization

The schemas for PSG harmonization were constructed by organizing information from eight PSG datasets into three components: biosignals, sleep parameters, and demographic features (Fig 1). The integration of biosignals was based on the signal acquisition manuals and sampling information provided by NSRR for each dataset. We organized biosignal attribute names and definitions and examined their inclusion across datasets. If a signal was recorded using a backup electrode rather than the recommended electrode, it was classified as the same signal measured from the recommended electrode. Furthermore, even for the same type of biosignal, structural and interpretive differences may result from the variations in electrode configuration, such as whether the signal was recorded using a unipolar or bipolar montage. Accordingly, in this study, such signals were considered as distinct attributes (Table 1). The EEG signals from ABC, CFS, MrOS, and SOF were recorded using a unipolar configuration (e.g., C3, C4). Instead, APPLE, SHHS, MESA, and WSC used a bipolar configuration (e.g., C3-M2, C4-A1). In unipolar EEG recordings, the electric potential at a specific site is measured relative to a reference electrode, whereas bipolar recordings represent the voltage difference between two electrodes and are regarded as distinct signals. For ECG signals, ABC, CFS, MrOS, and SOF provide unipolar recordings. Herein, the negative (-) and positive (+) electrodes were recorded as separate channels. In contrast, APPLE, MESA, SHHS, and WSC provided bipolar signals representing the voltage difference between the two electrodes. Similarly, for chin or submental EMG, certain datasets provided separate unipolar recordings from the left, right, and central chin or submental regions, whereas others provided bipolar derivations. Because chin or submental EMG is a core PSG signal used for sleep staging, particularly for identifying REM sleep based on muscle-tone information, these channels were explicitly included in the biosignal schema. Additional EMG channels recorded from other anatomical sites, such as limb EMG, were classified separately according to their anatomical location and clinical purpose, such as the assessment of limb movements or periodic limb movements.

**Table 1:** Dataset-wise configuration of the EEG, ECG, and EMG channels.

Dataset								
Signal	ABC	APPLE	CFS	MESA	MrOS	SHHS	SOF	WSC
**EEG**	F3, F4, C3, C4, O1, O2, M1, M2	C3-M2, C4-M1, O1-M2, O2-M1	C3, C4, M1, M2	C4-M1, Fz-Cz, Cz-Oz	C3, C4, A1 (M1), A2 (M2)	C3-A2, C4-A1	C3, C4, A1, A2	F3-M2, Fz-M2, C3-M2, Cz-M2, O1-M2, Pz-M2
**ECG**	ECG1, ECG2	ECG	ECG1, ECG2	EKG	ECG L (ECGL), ECG R (ECGR)	ECG	ECG1, ECG2	ECG
**Chin EMG**	Chin1, Chin2, Chin3	EMG	EMG1, EMG2, EMG3	EMG	L Chin (LChin), R Chin (RChin)	EMG	LChin, EMG/L, RChin, EMG/R	cchin_I, chin

Overview of EEG, ECG, and chin EMG channel configurations across the eight PSG datasets, including electrode placement and montage type (unipolar or bipolar). The variations in channel labels used for the same physiological signal across datasets are shown in parentheses. ABC: Apnea, Bariatric Surgery, and CPAP Study; APPLE: Apnea Positive Pressure Long-term Efficacy Study; CFS: Cleveland Family Study; MESA: Multi-Ethnic Study of Atherosclerosis; MrOS: Osteoporotic Fractures in Men Study; SHHS: the Sleep Heart Health Study; SOF: Study of Osteoporotic Fractures; WSC: Wisconsin Sleep Cohort; EEG: electroencephalogram; ECG: electrocardiogram; Chin EMG: chin electromyography.

**Fig 1:**
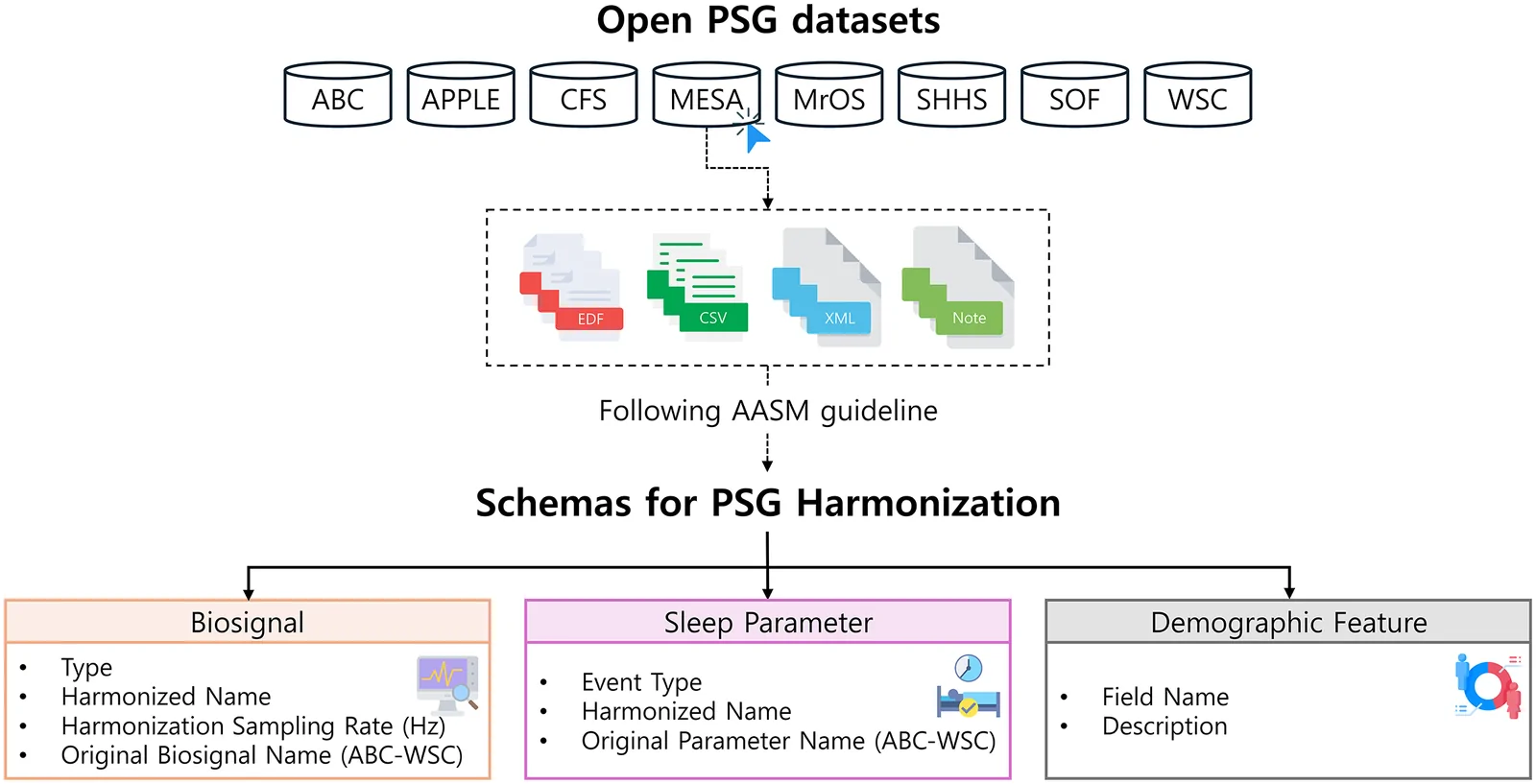
Workflow of the Schema Construction for PSG Harmonization. The schemas for PSG harmonization were devised by investigating the structure and content of EDF, XML, CSV, and documentation files from eight publicly available PSG datasets. Based on AASM standards, we defined unified schemas encompassing biosignals, sleep parameters, and demographic information. PSG: polysomnography; EDF: European data format; CSV: comma-separated values; XML: extensible markup language; AASM: American Academy of Sleep Medicine.

The harmonized naming system for biosignal attributes was developed by considering the signal type and recording configuration. When a given signal was present in a dataset, both the harmonized biosignal attribute name and the original signal label from the raw data were recorded.

The sampling rates of the biosignals included in the schema for biosignal harmonization varied across datasets even for the same biosignal. To address this, we defined a harmonization sampling rate for each signal to support consistent integration of previously collected heterogeneous PSG datasets. The harmonization sampling rates were determined by considering the sampling frequency distribution of the source datasets, relevant technical recommendations from the AASM, the physiological characteristics and clinical role of each signal in sleep analysis, and sampling rates used in previous AI-based sleep studies. When the same or similar sampling frequency was commonly observed across source datasets, that value was prioritized. When sampling frequencies varied across datasets, harmonization sampling rates were selected to minimize unnecessary resampling while preserving signal information relevant to PSG-based sleep analysis. Detailed signal-specific rationale for these rates is provided in S1 Appendix.

Biosignals were resampled to the corresponding harmonization sampling rates using a polyphase filtering-based resampling method [18]. This method performs sample-rate conversion by upsampling the signal, applying a zero-phase low-pass finite impulse response (FIR) filter, and then downsampling the filtered signal within an efficient polyphase structure. The low-pass filtering step suppresses image spectra introduced during upsampling and reduces aliasing that may occur during downsampling [19,20]. Because FIR filters can be designed with a linear-phase response, and because the implementation compensates for the associated filter delay, this approach is considered useful for reducing phase-related temporal shifts and preserving peak timing during resampling [19,21]. Polyphase implementation is a computationally efficient structure for filtering-based sample-rate conversion [22,23]. Related resampling procedures are also supported in established EEG and neurophysiological signal-processing software, including EEGLAB and MNE-Python [24–28]. Resampling was performed for sampling-rate harmonization and was not intended to recover physiological information absent from the original recordings.

The proposed harmonization framework focused on structural harmonization across datasets, including channel-name mapping, parameter harmonization, unit conversion, and resampling. Signal-level preprocessing procedures, such as artifact detection, artifact removal, denoising, and signal quality assessment, were excluded because the framework was designed to harmonize dataset structure rather than to modify the physiological signal content.

The sleep parameters for each dataset were reviewed using both the official documentation provided by NSRR and the actual variables present in the corresponding CSV files. The inclusion of each parameter was verified individually. The following documentation sources were used in this analysis: ABC, APPLE, CFS, MESA, MrOS, SHHS, SOF, and WSC [29–36]. In particular, sleep stages were validated using 30s annotation data recorded in XML files. The parameter harmonization was conducted based on the terminology used in the AASM guidelines. It involved reviewing the consistency of parameter names, the presence of missing or duplicated parameters, and the consistency of measurement units and structural formatting. For both biosignals and sleep parameters, the attribute names and units from the original EDF and CSV files were compared with the schemas for PSG harmonization.

In this study, we also investigated demographic and sleep-related clinical features commonly included across datasets. Key features, including race, sex, and age, were harmonized to ensure consistency in naming and encoding across datasets. Unit conversions were applied where necessary. For example, height and weight were converted from inches to centimeters and from pounds to kilograms, respectively. Obstructive sleep apnea (OSA) severity was categorized using age-specific apnea-hypopnea index (AHI) thresholds after separating records into adult and pediatric groups based on an age threshold of 18 years [37]. For adult records (age >18 years), OSA severity was classified as normal (AHI <5), mild (5≤ AHI <15), moderate (15≤ AHI <30), and severe (AHI ≥30) [38]. For pediatric records (age ≤ 18 years), OSA severity was classified as normal (AHI <1), mild (1≤ AHI <5), moderate (5≤ AHI <10), or severe (AHI ≥10) [39–41]. This derived sleep-related clinical feature was included to enhance clinical interpretability and support subgroup definition and stratified analyses in sleep research.

The schema construction and mapping process were conducted based on predefined harmonization criteria derived from the AASM guidelines, official NSRR documentation, and the dataset-specific metadata. Three researchers participated in the iterative review process to verify the consistency of biosignal, sleep parameter, and demographic feature harmonization across datasets. When ambiguities arose, the original dataset documentation, signal definitions, and parameter descriptions were re-examined and reviewed according to the predefined harmonization criteria. This review process also included structural ambiguities related to electrode configuration, such as the unipolar and bipolar montage distinctions described above. For example, some leg-related channels were described similarly as leg EMG in montage information but showed two distinct sampling-rate patterns across datasets, approximately 32–64 Hz and 128–256 Hz. To resolve this ambiguity, the source documentation and dataset-specific metadata were reviewed to confirm the recording site, sensor type, and measurement principle of each channel. Channels corresponding to EMG recordings were mapped as leg EMG, whereas channels derived from movement- or piezoelectric-based recordings were classified separately as movement-based limb signals. Accordingly, the harmonized biosignal names and harmonization sampling rates were assigned separately for these signal types. This mapping decision was made after collective review and discussion of the source materials by the three researchers. The resulting mappings were then incorporated into the final schemas.

### Validation of the integrated PSG dataset

To evaluate whether the integrated PSG dataset captures the demographic and sleep-related clinical diversity derived from multiple heterogeneous PSG cohorts, we conducted descriptive statistical analyses on key features including sex, age, body mass index (BMI), race, and OSA severity. To further assess whether the dataset supports the construction of structurally balanced test sets, stratified random sampling was performed in the adult population, defined as age >18 years, using sex, BMI category, and OSA severity. BMI was categorized using a threshold of 25 kg/m^2^ based on the World Health Organization (WHO) criteria, and OSA severity was stratified into four categories: normal, mild, moderate, and severe [38–42]. The restriction to adult records reflected the composition of the source datasets rather than a limitation of the proposed schema; records of individuals aged ≤18 years were concentrated in a limited number of source datasets (S1 Table) and were therefore less suitable for this repeated subgroup-based sampling test across heterogeneous cohorts. In the integrated dataset, 4,430 of 14,051 subjects (31.5%) contributed more than one sleep record. In the repeated sampling test, each sleep record was used as the sampling unit. One hundred sleep records were randomly selected from each subgroup combination defined by sex, BMI category, and OSA severity, and this procedure was repeated 10 times to evaluate the reproducibility of record-level test set composition across repeated samplings.

## Results

### Schemas for PSG harmonization

In this study, we proposed schemas for PSG harmonization to integrate PSG data collected from multiple centers. The aim was to harmonize structurally heterogeneous datasets. The schemas for PSG harmonization encompassed three components: biosignals, sleep parameters, and demographic features (Fig 1).

The schema for biosignal harmonization defined 59 biosignals. Each of these was annotated with its type, harmonized biosignal attribute name, and harmonization sampling rate, as detailed in S2 Table. The biosignal types were categorized into nine groups according to the AASM guidelines: EEG, EOG, ECG, leg EMG, chin EMG, airflow, respiratory effort, oxygen saturation (SpO_2_), and body position. Subject-derived physiological signals recorded as EDF signal channels that were not included in these predefined AASM-based core PSG biosignal categories were classified as “Other.” This category included heart rate. These nine categories are described in detail in S3 Table. The harmonized attribute names were designed to reflect the characteristics of each signal. For biosignals measured using electrodes (such as EEG, EOG, ECG, and EMG), the naming convention combined the signal type with the electrode configuration. This yielded names such as eeg_f3, ecg_1, and emg_leg. In contrast, signals that did not require spatial or positional differentiation (such as airflow, snoring, and thorax) were defined using a simple attribute name (e.g., thorax).

In the schema for biosignal harmonization, the presence of each signal across the eight PSG datasets was documented alongside its corresponding channel name as defined in the EDF. The harmonization sampling rates varied by signal category and were specified as follows: EEG = 128 Hz, EOG = 128 Hz, ECG = 128 Hz, chin EMG = 256 Hz, leg EMG = 256 Hz, limb movement signals = 64 Hz, snoring sound = 256 Hz, airflow, nasal pressure, thorax, abdomen, and respiratory effort signals = 32 Hz, plethysmography waveform = 128 Hz, and SpO_2_, pulse rate, body position, and heart rate = 1 Hz (S2 Table). Detailed signal-specific rationale for the harmonization sampling rates is provided in S1 Appendix.

The schema for sleep parameter harmonization proposes 122 sleep parameters, in conjunction with their event types and harmonized sleep parameter attribute names, as detailed in S4 Table. The event types were defined according to AASM guidelines and were categorized into five groups: sleep scoring, arousal, cardiac, body movement, and respiratory. These five categories are described in detail in S5 Table. Harmonized attribute names have been assigned to each parameter based on its definition. If the AASM provided a clear abbreviation (such as wake after sleep onset), we used the official abbreviation. If no official abbreviation had been provided by the AASM, we defined a new name that accurately reflected the parameter’s meaning. In the schema for sleep parameter harmonization, for each of the eight PSG datasets, the original name was documented as recorded in the CSV or an XML file when a given sleep parameter was present.

The schema for demographic feature harmonization consisted of nine attributes. Each of these was accompanied by a detailed description: subject ID, visit number, sex, age, race, BMI, weight, height, and OSA severity (Table 2). Race was categorized into seven groups. OSA severity was classified into four levels (normal, mild, moderate, and severe) based on age-specific AHI criteria [38–41].

**Table 2:** Schema for demographic feature harmonization.

Attribute name	Description
subject_id	A unique subject identifier used to distinguish individual participants
visit_num	A numerical identifier used to distinguish multiple visits from the same subject
sex	Sex (Male/Female)
age	Age of the subject at the time of the PSG recording
race	Race information includes the following categories: White, Black or African American, Hispanic, Asian, American Indian or Alaska Native, Multiple, Other, and Not Reported
bmi	Body mass index (kg/m^2^)
weight	Body weight (standardized to kilograms)
height	Height (standardized to centimeters)
osa_severity	OSA severity based on age-specific apnea–hypopnea index (AHI) thresholds: For adult records (age > 18 years), OSA severity was categorized as Normal (AHI < 5), Mild (5 ≤ AHI < 15), Moderate (15 ≤ AHI < 30), and Severe (AHI ≥ 30). For pediatric records (age ≤ 18 years), OSA severity was categorized as Normal (AHI < 1), Mild (1 ≤ AHI < 5), Moderate (5 ≤ AHI < 10), and Severe (AHI ≥ 10).

Demographic attributes included in the harmonization schema and their definitions.

### Integrated PSG dataset

We constructed an integrated PSG dataset by applying schemas for the PSG harmonization of biosignals, sleep parameters, and demographic features to eight publicly available PSG datasets (Fig 2). The integrated dataset consisted of the following three components:

**Fig 2:**
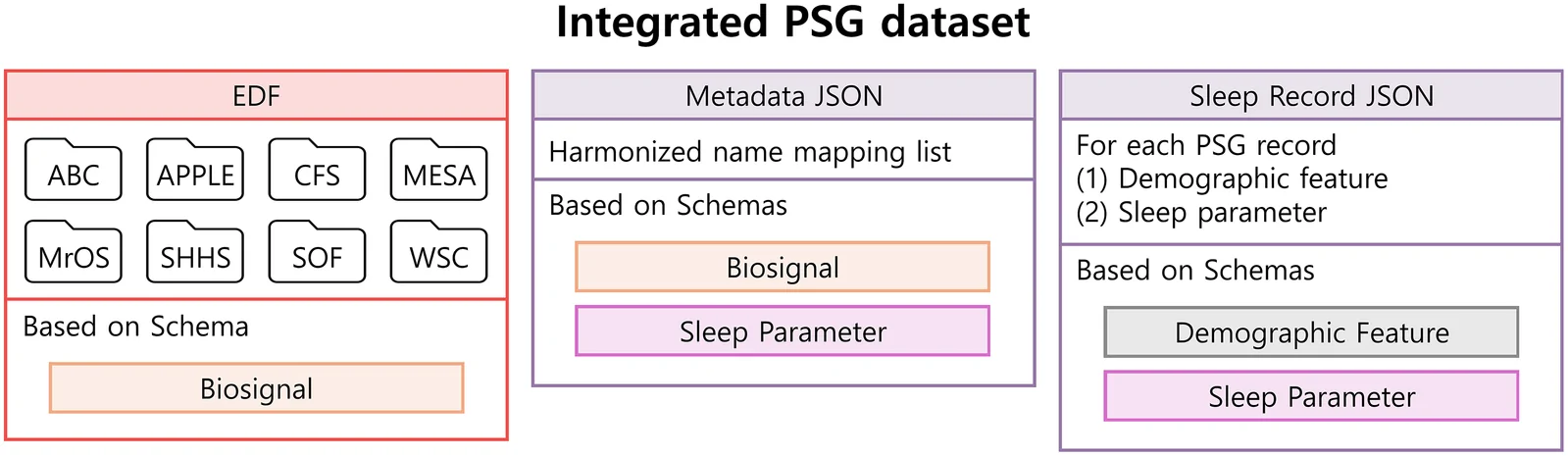
Integrated PSG dataset based on schemas for PSG harmonization. Three schema components were combined selectively to generate an integrated dataset composed of biosignal EDF files and sleep parameter JSON files. PSG: polysomnography; EDF: European data format; JSON: JavaScript object notation.

- Harmonized biosignal file: Available biosignal channels from each dataset were converted into a harmonized structure with unified labels and sampling rates according to the schema for biosignal harmonization. The harmonized signals were subsequently stored in EDF files.
- Metadata file for biosignal and sleep parameter composition: The list of biosignals and sleep parameters included in each dataset was mapped to the harmonized attribute names defined in the schemas. These metadata files were stored in the JavaScript object notation (JSON) format.
- Sleep record information file: The demographic features and sleep parameters for each sleep record were organized according to the harmonized attribute names and descriptions defined in the schemas and were stored in the JSON format. If a subject had multiple sleep records, each was saved as a separate entry distinguished by a visit_num field. Only available demographic variables and sleep parameters were included for each sleep record, whereas sleep records were excluded only when essential identifiers such as EDF files or subject identifiers were unavailable.

The integrated PSG dataset included a total of 19,170 sleep records. The demographic characteristics of the included subjects can be summarized as follows: the sex distribution is 58.8% male and 41.2% female. The age distribution is 65.8 ± 13.4, with the ages ranging from 6–90 years. The race distribution shows that the dataset was composed predominantly of White (80.8%), Black (10.1%), Hispanic (3.2%), and Asian (2.4%) individuals. The racial composition was comparable to that reported in clinical cohort studies conducted in the United States [43]. The BMI distribution was 28.9 ± 5.9 kg/m^2^, which fell within the overweight category according to the WHO classification [44]. The distribution of OSA severity was as follows: normal (12.5%), mild (27.4%), moderate (22.6%), and severe (18.6%) [38]. Overall, the integrated PSG dataset captured variability across several demographic and clinical characteristics, including sex, age, race, BMI, and OSA severity, through the integration of multiple heterogeneous PSG cohorts. Descriptive statistics for the dataset are presented in Table 3.

**Table 3:** Demographic characteristics of the integrated polysomnography dataset.

Demographics	Summary statistics
Total (n)	19,170
Sex (n, %)	
Male	11,263 (58.8%)
Female	7,907 (41.2%)
Age (years)	65.8 ± 13.4 [6.8–90.0]
BMI (kg/m²)	28.9 ± 5.9 [13.3–84.8]
Race (n, %)	
White	15,490 (80.8%)
Black or African American	1,942 (10.1%)
Hispanic	607 (3.2%)
Other	579 (3.0%)
Asian	451 (2.4%)
American Indian or Alaska native	41 (0.2%)
Multiple	35 (0.2%)
Not reported	25 (0.1%)
OSA severity (n, %)	
Normal	2,389 (12.5%)
Mild	5,248 (27.4%)
Moderate	4,338 (22.6%)
Severe	3,561 (18.6%)
Unknown	3,634 (19.0%)

Summary statistics of key demographic and clinical characteristics in the integrated PSG dataset. OSA: Obstructive sleep apnea. The continuous attributes are presented as mean ± standard deviation, with the range in parentheses. The categorical attributes are shown as counts and percentages.

### Sampling reproducibility test

Fig 3 shows the demographic characteristics and OSA severity distribution of test sets sampled 10 times from the integrated PSG dataset, demonstrating the reproducibility of the test set composition. For the predefined stratification variables, the proportion of sleep records from female participants and sleep records with BMI ≥ 25 kg/m^2^ remained fixed at 50.0% across the 10 iterations. The four OSA severity categories were also evenly represented, with sleep records classified as normal, mild, moderate, and severe each accounting for 25.0% of the sampled sleep records in each iteration. No variability was observed across iterations for the distributions of sex, BMI category, or OSA severity. The mean age showed minimal variation across repeated samples, with a coefficient of variation (CV) of 0.52%. Although race was not used as a stratification variable, the racial composition of the sampled sleep records remained stable across repeated samples while reflecting the distribution of the integrated adult dataset: the CVs were 1.01% for records from White participants and 4.21% for records from Black participants.

**Fig 3:**
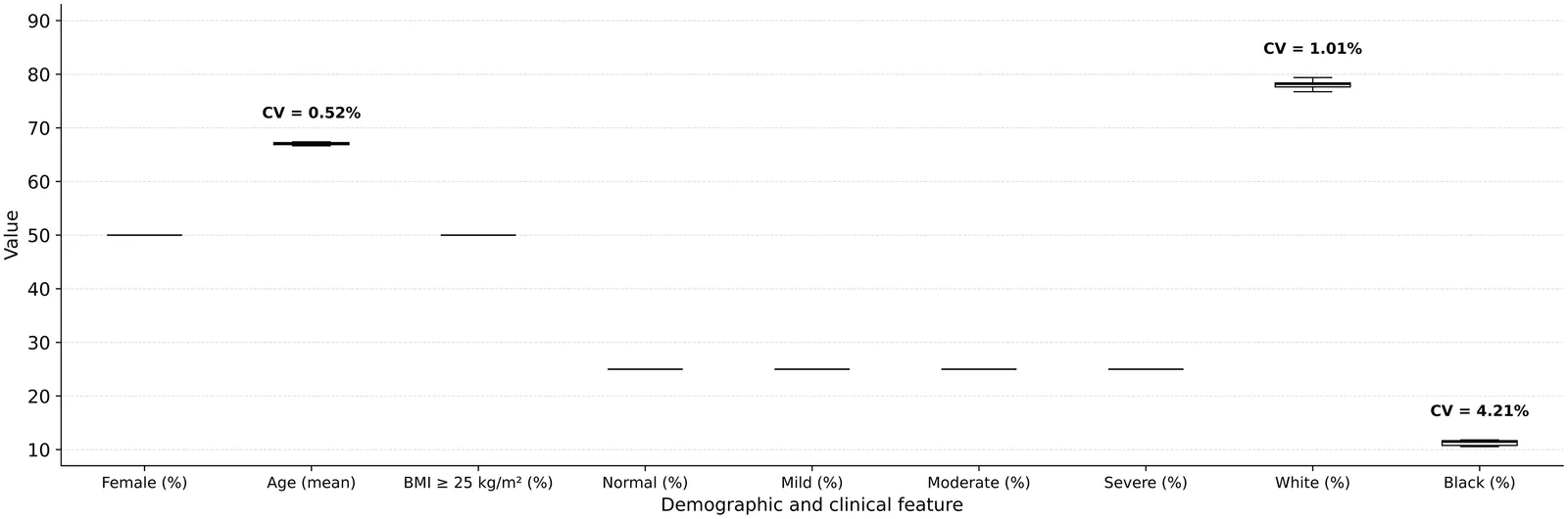
Box plot of demographic and clinical features across 10 sampling trials. Sex is represented by the proportion of sleep records from female participants (%), age indicates the sample mean in years, and body mass index is represented by the proportion of sleep records with BMI ≥ 25 kg/m^2^ (%). Obstructive sleep apnea severity is shown as the percentage of sampled sleep records classified into four categories (Normal, Mild, Moderate, or Severe) in each iteration. Sex, BMI category, and obstructive sleep apnea severity correspond to the predefined stratification variables used in the repeated sampling test. Race is represented by the proportions of sleep records from White participants (%) and Black participants (%). Non-zero coefficient of variation (CV, %) values are annotated above boxes; values rounded to 0.00% are not shown.

## Discussion

This study proposes schemas for PSG harmonization that enable integration notwithstanding the structural heterogeneity in PSG data collected from multiple centers. These schemas support the consistent use of different types of information, including biosignals and sleep parameters.

The proposed schemas are based on the AASM guidelines and harmonize the differences in structure, terminology, and units across publicly available PSG datasets. It incorporates a unified framework encompassing 59 biosignals, 122 sleep parameters, and 9 demographic features. In addition, mapping information was constructed by organizing dataset-specific presence and naming differences for each attribute. This would enable researchers to efficiently select relevant attributes for multi-center analyses.

To validate the practical applicability of the proposed schemas, this study constructed an integrated PSG dataset by combining eight publicly available PSG datasets based on these schemas. The schemas unify the attribute names and units of heterogeneous biosignals and sleep parameters. This enables researchers to utilize multi-center data without requiring dataset-specific preprocessing. This, in turn, would reduce the preprocessing load and enhance the efficiency of algorithm evaluation. Furthermore, in a repeated sampling experiment using stratified random sampling by sex, BMI category, and OSA severity, the sampled sleep records maintained the intended balance across the predefined strata in all 10 iterations. This result reflects the application of the predefined stratification scheme and indicates that the integrated dataset can be used to construct record-level test subsets according to predefined demographic and sleep-related clinical strata. The mean age showed minimal variation across repeated samples, and the racial composition of the sampled sleep records remained stable despite race not being used as a stratification variable. This consistency suggests that the integrated dataset can support reproducible construction of structurally balanced test subsets for future multi-cohort model evaluation and benchmarking. Because the repeated sampling test used sleep records as the sampling unit, records from the same subject could appear in different repeated test sets when multiple records were available for that subject. This repeated sampling test was conducted to assess the reproducibility of record-level test-set composition and did not involve model training, train/test splitting, or cross-validation. Therefore, this setting should not be interpreted as a subject-level model validation design. The demographic schema includes subject ID and visit number, allowing researchers to apply subject-level splitting, visit-level splitting, or exclusion of repeated records according to the purpose of their analysis. Subject-level splitting is recommended when such test subsets are used for model evaluation. It ultimately facilitates the development of benchmarking frameworks, thereby enhancing the utility of the dataset in sleep research.

However, the proposed schemas for PSG harmonization have several limitations that should be acknowledged. First, although the harmonization sampling rates were selected to support PSG-based sleep analysis and multi-cohort AI model benchmarking, the harmonized signals may not be suitable for analyses requiring preservation of fine-scale high-frequency waveform morphology. For example, detailed ECG morphology analysis, fine-scale plethysmography waveform or peak analysis, and high-frequency EMG characterization may require access to native higher-rate recordings. The present framework was designed primarily for sleep-analysis contexts in which ECG is used for heart rate or rhythm-related information, PSG-derived signals are used for oxygen saturation or pulse-related monitoring, and EMG or limb channels are used for muscle-tone or event-level movement interpretation.

Second, signal-level quality control was not incorporated into the present harmonization framework. We recognize that variability in signal quality, including artifacts, lead-off events, noise, and channel-specific recording problems, is an important issue when integrating real-world PSG datasets. However, artifact detection, denoising, lead-off detection, and exclusion of noisy recordings require signal-specific and often task-specific criteria and may involve modifying or excluding physiological signal content. Because the present study focused on schema-level harmonization, including channel-name mapping, parameter harmonization, unit conversion, and sampling-rate harmonization, these signal-level quality control procedures were excluded. Future studies should extend the harmonization framework by incorporating standardized signal quality assessment modules or signal quality metadata to support downstream model evaluation and benchmarking.

Third, the schemas were developed primarily based on publicly available PSG datasets collected from adult populations. Consequently, they do not include certain respiratory signals and sleep parameters specific to pediatric sleep studies, such as end-tidal CO_2_. This indicates that when integrating datasets focused on specific populations such as pediatric populations, additional signals or parameters may need to be supplemented or extended. In addition, some publicly available PSG datasets include questionnaire-based or medical history-related variables, such as medication use, smoking status, and comorbidities. Future studies should explore the integration of such variables into the harmonization framework. Separately, although the integrated dataset combines multiple publicly available PSG cohorts, its demographic composition reflects the source datasets used for integration and remains predominantly composed of older adult and White populations. As summarized in S1 Table, records from individuals aged ≤18 years were also concentrated in a limited number of source datasets. These patterns represent limitations of the resulting integrated dataset and the underlying source dataset composition, rather than limitations imposed by the proposed schema. Future studies may further improve population representativeness by incorporating datasets with broader age ranges and more diverse racial and ethnic backgrounds.

Fourth, although video recording is commonly performed in PSG for the evaluation of abnormal nocturnal behaviors and movement-related sleep disorders, synchronized video information was not considered in the present harmonization framework. Fifth, during the construction process of the schemas, the biosignals and sleep parameters, such as attribute names, definitions, and sampling rates, were mapped manually for each dataset. Therefore, integrating new datasets into the schemas or adding new signals and parameters may still require manual review and refinement in the initial stages.

## Conclusions

This study developed a schema-level harmonization framework for integrating heterogeneous biosignals, sleep parameters, and demographic and sleep-related clinical features across publicly available PSG datasets. By formalizing a unified structural specification aligned with established clinical guidelines, the proposed schemas reduce structural ambiguity across datasets and provide a systematic basis for consistent interpretation, structured integration, and reproducible algorithm evaluation across public PSG resources such as NSRR.

PSG data are inherently complex and comprise multi-channel physiological signals, heterogeneous acquisition settings, and guideline-dependent scoring definitions. Compared with studies based on single biosignals, PSG research requires formal structural and semantic organization before datasets can be jointly used for large-scale AI-based sleep research. The proposed framework provides a data-infrastructural basis for the reproducible use of public PSG repositories by harmonizing biosignal, sleep parameter, and demographic, and sleep-related clinical feature structures across heterogeneous datasets. Building on the harmonized dataset developed in this study, we are currently developing a platform for evaluating and benchmarking sleep AI models across multi-cohort PSG data. Future work should also extend the schema to incorporate more diverse populations and signal types, consider formal schema version-control strategies to accommodate updates to AASM guidelines and scoring definitions, and explore scalable methods for semi-automated schema expansion to support broader practical application.

## Supporting information

S1 Appendix Rationale for Signal-Specific Harmonization Sampling Rates. (DOCX)

S1 Table Overview of source datasets used for schema development. (DOCX)

S2 Table Schema for biosignal harmonization. (DOCX)

S3 Table Harmonized biosignal attribute names and descriptions. (DOCX)

S4 Table Schema for sleep parameter harmonization. (DOCX)

S5 Table Harmonized sleep parameter attribute names and descriptions. (DOCX)

## References

[1] Hirshkowitz M. The history of polysomnography: tool of scientific discovery. Sleep Medicine. Springer New York. 2015. p. 91–100. doi: 10.1007/978-1-4939-2089-1_13

[2] Zhang G-Q, Cui L, Mueller R, Tao S, Kim M, Rueschman M, et al. The National Sleep Research Resource: towards a sleep data commons. J Am Med Inform Assoc. 2018;25(10):1351–8. doi: 10.1093/jamia/ocy064 29860441 PMC6188513

[3] Azarbarzin A, Sands SA, Younes M, Taranto-Montemurro L, Sofer T, Vena D, et al. The sleep apnea-specific pulse-rate response predicts cardiovascular morbidity and mortality. Am J Respir Crit Care Med. 2021;203(12):1546–55. doi: 10.1164/rccm.202010-3900OC 33406013 PMC8483223

[4] Querejeta Roca G, Redline S, Punjabi N, Claggett B, Ballantyne CM, Solomon SD, et al. Sleep apnea is associated with subclinical myocardial injury in the community. The ARIC-SHHS study. Am J Respir Crit Care Med. 2013;188(12):1460–5. doi: 10.1164/rccm.201309-1572OC 24156237 PMC3917380

[5] Yeboah J, Redline S, Johnson C, Tracy R, Ouyang P, Blumenthal RS, et al. Association between sleep apnea, snoring, incident cardiovascular events and all-cause mortality in an adult population: MESA. Atherosclerosis. 2011;219(2):963–8. doi: 10.1016/j.atherosclerosis.2011.08.021 22078131 PMC3255297

[6] Goldberger AL, Amaral LA, Glass L, Hausdorff JM, Ivanov PC, Mark RG. PhysioBank, PhysioToolkit, and PhysioNet: components of a new research resource for complex physiologic signals. Circulation. 2000;101(23):e215–20. 10.1161/01.cir.101.23.e215 10851218

[7] BaHammam AS, Chee MW. Publicly available health research datasets: opportunities and responsibilities. Taylor & Francis. 2022. 10.2147/NSS.S390292 PMC9527360 36199429

[8] Strøm J, Engholm AL, Lorenzen KP, Mikkelsen KB. Common sleep data pipeline for combined data sets. PLoS One. 2024;19(8):e0307202. doi: 10.1371/journal.pone.0307202 39106236 PMC11302895

[9] Bakker JP, Tavakkoli A, Rueschman M, Wang W, Andrews R, Malhotra A, et al. Gastric banding surgery versus continuous positive airway pressure for obstructive sleep apnea: a randomized controlled trial. Am J Respir Crit Care Med. 2018;197(8):1080–3. doi: 10.1164/rccm.201708-1637LE 29035093 PMC5909166

[10] Blackwell T, Yaffe K, Ancoli-Israel S, Redline S, Ensrud KE, Stefanick ML, et al. Associations between sleep architecture and sleep-disordered breathing and cognition in older community-dwelling men: the Osteoporotic Fractures in Men Sleep Study. J Am Geriatr Soc. 2011;59(12):2217–25. doi: 10.1111/j.1532-5415.2011.03731.x 22188071 PMC3245643

[11] Chen X, Wang R, Zee P, Lutsey PL, Javaheri S, Alcántara C, et al. Racial/ethnic differences in sleep disturbances: the Multi-Ethnic Study of Atherosclerosis (MESA). Sleep. 2015;38(6):877–88. doi: 10.5665/sleep.4732 25409106 PMC4434554

[12] Quan SF, Chan CS, Dement WC, Gevins A, Goodwin JL, Gottlieb DJ, et al. The association between obstructive sleep apnea and neurocognitive performance--the Apnea Positive Pressure Long-term Efficacy Study (APPLES). Sleep. 2011;34(3):303-314B. doi: 10.1093/sleep/34.3.303 21358847 PMC3041706

[13] Quan SF, Howard BV, Iber C, Kiley JP, Nieto FJ, O’Connor GT, et al. The Sleep Heart Health Study: design, rationale, and methods. Sleep. 1997;20(12):1077–85. doi: 10.1093/sleep/20.12.1077 9493915

[14] Redline S, Tishler PV, Tosteson TD, Williamson J, Kump K, Browner I, et al. The familial aggregation of obstructive sleep apnea. Am J Respir Crit Care Med. 1995;151(3 Pt 1):682–7. doi: 10.1164/ajrccm/151.3_Pt_1.682 7881656

[15] Spira AP, Blackwell T, Stone KL, Redline S, Cauley JA, Ancoli-Israel S, et al. Sleep-disordered breathing and cognition in older women. J Am Geriatr Soc. 2008;56(1):45–50. doi: 10.1111/j.1532-5415.2007.01506.x 18047498

[16] Young T, Palta M, Dempsey J, Peppard PE, Nieto FJ, Hla KM. Burden of sleep apnea: rationale, design, and major findings of the Wisconsin Sleep Cohort study. WMJ. 2009;108(5):246–9. 19743755 PMC2858234

[17] Berry RB, Brooks R, Gamaldo C, Harding SM, Lloyd RM, Quan SF. AASM scoring manual updates for 2017 (version 2.4). American Academy of Sleep Medicine. 2017. 10.5664/jcsm.6576 PMC5406946 28416048

[18] SciPy community. SciPy API reference: scipy.signal.resample_poly. [cited in 2026 June 19]. https://docs.scipy.org/doc/scipy/reference/generated/scipy.signal.resample_poly.html#scipy.signal.resample_poly

[19] Bregovic R, Yu YJ, Saramaki T, Lim YC. Implementation of linear-phase FIR filters for a rational sampling-rate conversion utilizing the coefficient symmetry. IEEE Trans Circuits Syst I. 2011;58(3):548–61. doi: 10.1109/tcsi.2010.2072350

[20] Crochiere RE, Rabiner LR. Interpolation and decimation of digital signals—a tutorial review. Proc IEEE. 1981;69(3):300–31. doi: 10.1109/proc.1981.11969

[21] Widmann A, Schröger E, Maess B. Digital filter design for electrophysiological data--a practical approach. J Neurosci Methods. 2015;250:34–46. doi: 10.1016/j.jneumeth.2014.08.002 25128257

[22] Bellanger M, Bonnerot G, Coudreuse M. Digital filtering by polyphase network: application to sample-rate alteration and filter banks. IEEE Trans Acoust, Speech, Signal Process. 1976;24(2):109–14. doi: 10.1109/tassp.1976.1162788

[23] Zeineddine A, Nafkha A, Paquelet S, Moy C, Jezequel PY. Comprehensive survey of FIR-based sample rate conversion. J Sign Process Syst. 2020;93(1):113–25. doi: 10.1007/s11265-020-01575-6

[24] Braindecode Developers. Braindecode API reference: braindecode.preprocessing.Resample. [cited in 2026 June 19]. https://braindecode.org/dev/generated/braindecode.preprocessing.Resample.html

[25] MNE Developers. MNE API reference: mne.filter.resample. [cited in 2026 June 19]. https://mne.tools/stable/generated/mne.filter.resample.html

[26] NeuroKit2 Developers. NeuroKit2 signal processing API reference: signal_resample. [cited in 2026 June 19]. https://neuropsychology.github.io/NeuroKit/functions/signal.html#neurokit2.signal_resample

[27] EEGLAB. EEGLAB tutorial: changing the data sampling rate. [cited in 2026 June 19]. https://eeglab.org/tutorials/05_Preprocess/resampling.html

[28] The MathWorks, Inc. MATLAB signal processing reference: resample. [cited in 2026 June 19]. https://au.mathworks.com/help/signal/ref/resample.html

[29] National Sleep Research Resource. ABC dataset documentation. National Sleep Research Resource (NSRR). 2020. [cited in 2026 Febr 12]. https://www.sleepdata.org/datasets/abc

[30] National Sleep Research Resource. APPLES dataset documentation. National Sleep Research Resource (NSRR). 2023. [cited in 2026 Febr 12]. https://sleepdata.org/datasets/apples

[31] National Sleep Research Resource. CFS dataset documentation. National Sleep Research Resource (NSRR). 2020. [cited 2026 Febr 12]. https://www.sleepdata.org/datasets/cfs

[32] MESA dataset documentation. National Sleep Research Resource (NSRR). 2020. [cited in 2026 Febr 12]. https://sleepdata.org/datasets/mesa

[33] National Sleep Research Resource. MrOS dataset documentation. National Sleep Research Resource (NSRR). 2020. [cited in 2026 Febr 12]. https://sleepdata.org/datasets/mros

[34] National Sleep Research Resource. SHHS dataset documentation. National Sleep Research Resource (NSRR). 2020. [cited in 2026 Febr 12]. https://sleepdata.org/datasets/shhs

[35] National Sleep Research Resource. Sof dataset documentation. 2020. https://sleepdata.org/datasets/sof

[36] National Sleep Research Resource. WSC dataset documentation. National Sleep Research Resource (NSRR). 2025. [cited in 2026 Febr 12]. https://sleepdata.org/datasets/wsc

[37] Paruthi S, Brooks LJ, D’Ambrosio C, Hall WA, Kotagal S, Lloyd RM, et al. Recommended amount of sleep for pediatric populations: a consensus statement of the american academy of sleep medicine. J Clin Sleep Med. 2016;12(6):785–6. doi: 10.5664/jcsm.5866 27250809 PMC4877308

[38] Sleep–related breathing disorders in adults: recommendations for syndrome definition and measurement techniques in clinical research. Sleep. 1999;22(5):667–89. doi: 10.1093/sleep/22.5.667 10450601

[39] Kaditis AG, Alonso Alvarez ML, Boudewyns A, Alexopoulos EI, Ersu R, Joosten K, et al. Obstructive sleep disordered breathing in 2- to 18-year-old children: diagnosis and management. Eur Respir J. 2016;47(1):69–94. doi: 10.1183/13993003.00385-2015 26541535

[40] Marcus CL, Brooks LJ, Draper KA, Gozal D, Halbower AC, Jones J, et al. Diagnosis and management of childhood obstructive sleep apnea syndrome. Pediatrics. 2012;130(3):e714-55. doi: 10.1542/peds.2012-1672 22926176

[41] American Academy of Sleep Medicine. International classification of sleep disorders—third edition (ICSD-3). AASM Resour Libr. 2014;281:2313.

[42] World Health Organization. WHO guidelines on physical activity and sedentary behaviour. Geneva: World Health Organization. 2020. 33369898

[43] Turner BE, Steinberg JR, Weeks BT, Rodriguez F, Cullen MR. Race/ethnicity reporting and representation in US clinical trials: a cohort study. Lancet Reg Health Am. 2022;11:100252. doi: 10.1016/j.lana.2022.100252 35875251 PMC9302767

[44] World Health Organization. Obesity: preventing and managing the global epidemic: report of a WHO consultation. World Health Organization. 2000. 11234459

