# Supplementary material for: Polysomnography harmonization schemes: A multi-institutional sleep research resource
Source: PLoS One. 2026 Nov 5;21(11):e0361116. doi: 10.1371/journal.pone.0361116 (PMC13739147; doi:10.1371/journal.pone.0361116)
Supplement: Supplementary file 1 [file pone.0361116.s001.docx]

**Supplementary Appendix1. Rationale for Signal-Specific Harmonization Sampling Rates**

The harmonization sampling rates in this study were defined to support the integration of previously collected heterogeneous polysomnography (PSG) datasets. These sampling rates should not be interpreted as acquisition-level sampling recommendations for newly recorded PSG studies. Instead, they were selected to provide a common structure for integrating legacy PSG datasets collected using different recording systems, sensor configurations, and sampling frequencies.

The sampling rates were determined by considering four factors: the sampling frequency distribution of the source datasets, relevant technical recommendations from the American Academy of Sleep Medicine (AASM), the physiological characteristics and clinical role of each signal in PSG-based sleep analysis, and sampling-rate harmonization practices used in previous artificial intelligence (AI)-based sleep studies [1]. When the same or a similar sampling frequency was commonly observed across source datasets, that value was prioritized. When sampling frequencies varied substantially across datasets, the sampling rate was selected based on the above considerations, with the aim of reducing unnecessary resampling.

1) Electroencephalography, electrooculography, and electrocardiography

For electroencephalography (EEG), electrooculography (EOG), and electrocardiography (ECG), the harmonization sampling rate was set to 128 Hz. Across the source datasets, these signals were recorded at heterogeneous sampling frequencies, including 50 Hz for some EOG channels and values of ~100, 125, 128, 200, and 256 Hz depending on the dataset and channel configuration [2-10]. The 128-Hz sampling rate was selected by considering the sampling frequency distribution of the source datasets and the frequency ranges in which clinically relevant physiological information for PSG-based sleep analysis is typically represented while reducing unnecessary resampling across heterogeneous datasets.

For EEG, the major spectral features used in PSG-based sleep staging are largely represented below ~30 Hz, including delta, theta, alpha, beta, and sleep spindle activities [1]. For EOG, the clinically relevant information in PSG-based sleep staging is the identification of ocular movement events, including slow eye movements, rapid eye movements associated with rapid eye movement (REM) sleep, and eye blinks, all of which are characterized by temporal patterns of several hundred milliseconds or longer [1]. The main frequency content of EOG signals is generally reported to be concentrated within ~0–30 Hz, with some references extending this range to ~50 Hz [11-13].

For ECG, the clinically relevant information in PSG-based sleep analysis is primarily heart rate and cardiac rhythm information, including bradycardia, tachycardia, sleep-associated rhythm disturbances, and QRS-complex-based rhythm assessment [1]. The QRS complex exhibits major spectral energy at ~5–25 Hz, mostly below ~40 Hz; similarly, the Pan–Tompkins algorithm, a representative QRS detection method, uses a 5–15 Hz band-pass range for QRS detection [14, 15]. In the context of ECG monitoring focusing on heart rate and arrhythmia assessment, a bandwidth of ~0.5–50 Hz is commonly used [16].

Taken together, the upper frequency range considered for PSG-relevant EEG, EOG, and ECG information was ~50 Hz. Based on the Nyquist criterion, preserving frequency content up to 50 Hz requires a sampling rate of at least 100 Hz. The 128-Hz harmonization sampling rate provides a Nyquist frequency of 64 Hz, leaving a margin above 50 Hz for anti-alias filter roll-off during resampling. Therefore, 128 Hz was considered an appropriate practical sampling for harmonizing EEG, EOG, and ECG in the context of PSG-based sleep analysis.

2) Chin electromyography

For chin electromyography (EMG), the harmonization sampling rate was set to 256 Hz. Chin EMG is used in PSG-based sleep staging to evaluate muscle tone, particularly during REM sleep, and the AASM recommends a minimum sampling rate of 200 Hz for EMG signals [1]. Considering the 128–256 Hz range observed in the source datasets and the use of 256 Hz in a prior chin EMG study during REM sleep [17], 256 Hz was selected as the harmonization sampling rate, thereby satisfying the AASM minimum sampling recommendation.

3) Leg EMG

For leg EMG, the harmonization sampling rate was set to 256 Hz. Leg EMG is primarily used in PSG to evaluate periodic limb movement (PLM)-related events, for which the key information includes event occurrence, timing, duration, and repetition [1]. Because leg EMG represents electromyographic activity and the AASM recommends a minimum sampling rate of 200 Hz for EMG signals, 256 Hz was selected as the harmonization sampling rate.

4) Limb movement

A separate harmonization sampling rate of 64 Hz was assigned to movement-based limb signals. Some public PSG datasets include leg-related channels in European Data Format files measured using piezoelectric sensors and recorded at lower sampling frequencies, such as 32–64 Hz [4-6, 8]. Because piezoelectric limb movement signals capture leg movement or vibration rather than muscle electrical activity, the EMG sampling criterion was not directly applied to these channels. A prior study also used leg activity signals sampled at 40 Hz and represented as lower-rate amplitude signals for PLM and periodic leg movement during wakefulness assessment [18]. Accordingly, 64 Hz was selected for movement-based limb signals to preserve event-level movement information while avoiding unnecessary conversion to EMG-level sampling rates.

5) Airflow, nasal pressure, thorax, abdomen, and respiratory effort

For airflow, nasal pressure, thorax, abdomen, and respiratory effort, the harmonization sampling rate was set to 32 Hz. In the source datasets, these respiratory signals were commonly recorded at 16 Hz or 32 Hz, whereas the AASM recommends a minimum sampling rate of 25 Hz for respiratory signals [1-9]. Thus, 32 Hz was selected because it satisfies the AASM minimum recommendation and corresponds to a sampling rate observed in multiple public PSG datasets.

6) Snoring

For snoring sounds, the harmonization sampling rate was set to 256 Hz. Snoring sound channels are used in PSG to evaluate snoring occurrence and vibration-related information, and several source datasets recorded snoring-related signals at 256 Hz [1-4, 9]. Because the AASM recommends a minimum sampling rate of 200 Hz for snoring sound signals, 256 Hz was selected as the harmonization sampling rate [1].

7) Plethysmography

For plethysmography (PPG), the harmonization sampling rate was set to 128 Hz. Plethysmography waveform channels provide pulse waveform information used as the basis for deriving oxygen saturation (SpO₂) and pulse rate and are therefore distinct from derived numerical SpO₂ or pulse rate variables. In the source datasets, plethysmography waveform channels were commonly recorded within the range of 128–256 Hz, and prior studies have recommend sampling rates of ≥100 Hz for reliable pulse waveform analysis. Technical documentation and product information for commercial sleep-analysis or pulse oximetry systems, including devices cleared by the U.S. Food and Drug Administration, also report PPG sampling rates of ≥100 Hz [2, 4, 5, 19-22]. Therefore, 128 Hz was selected as the harmonization sampling rate for plethysmography waveform signals.

8) SpO_2_, pulse rate, body position, heart rate

For SpO₂, pulse rate, body position, and heart rate, the harmonization sampling rate was set to 1 Hz because these variables are slowly varying numerical or categorical signals and were commonly represented at 1 Hz in the source PSG datasets [2-9].

Resampling to a common sampling rate has been widely used in sleep dataset integration and AI-based sleep studies to manage heterogeneous PSG signals. For example, U-Sleep resampled EEG and EOG signals to 128 Hz, SleepFM standardized PSG signals to 128 Hz, SleepMaMi resampled signals to 100 Hz, DRIVEN harmonized signals to 64 Hz, and PFTSleep resampled signals to 125 Hz [23-28]. These studies indicate that common-rate resampling is a routine preprocessing step for integrating heterogeneous PSG datasets and developing cross-dataset sleep models. The resampling procedure in the present study was applied in the same harmonization context, after the signal-specific harmonization sampling rates had been defined. Resampling was performed to align sampling frequencies across datasets and was not intended to recover physiological information that was absent from the original recordings.

**Supplementary References**

1. Berry RB, Brooks R, Gamaldo C, Harding SM, Lloyd RM, Quan SF, et al. AASM scoring manual updates for 2017 (version 2.4). American Academy of Sleep Medicine; 2017. p. 665-6.

2. National Sleep Research Resource. ABC dataset documentation. 2020 [cited 2026 Feb 12]. In: National Sleep Research Resource (NSRR) [Internet]. Available from: https://www.sleepdata.org/ datasets/abc.

3. National Sleep Research Resource. APPLE dataset documentation. 2023 [cited 2026 Feb 12]. In: National Sleep Research Resource (NSRR) [Internet]. Available from: https://sleepdata.org/ datasets/apples.

4. National Sleep Research Resource. CFS dataset documentation. 2020. [cited 2026 Feb 12]. In: National Sleep Research Resource (NSRR) [Internet]. Available from: https://www.sleepdata.org/ datasets/cfs.

5. National Sleep Research Resource. MESA dataset documentation. 2020. [cited 2026 Feb 12]. In: National Sleep Research Resource (NSRR) [Internet]. Available from: https://sleepdata.org/ datasets/mesa.

6. National Sleep Research Resource. MrOS dataset documentation. 2020. [cited 2026 Feb 12]. In: National Sleep Research Resource (NSRR) [Internet]. Available from: https://sleepdata.org/ datasets/mros.

7. National Sleep Research Resource. SHHS dataset documentation. 2020. [cited 2026 Feb 12]. In: National Sleep Research Resource (NSRR) [Internet]. Available from: https://sleepdata.org/ datasets/shhs.

8. National Sleep Research Resource. SOF dataset documentation. 2020. [cited 2026 Feb 12]. In: National Sleep Research Resource (NSRR) [Internet]. Available from: https://sleepdata.org/ datasets/sof.

9. National Sleep Research Resource. WSC dataset documentation. 2025 [cited 2026 Feb 12]. In: National Sleep Research Resource (NSRR) [Internet]. Available from: https://sleepdata.org/ datasets/wsc.

10. National Sleep Research Resource. National Sleep Research Resource [Internet]. 2025 [cited 2025 Aug 15]. Available from: https://sleepdata.org/.

11. Constable PA, Bach M, Frishman LJ, Jeffrey BG, Robson AG, org ISfCEoVhwi. ISCEV Standard for clinical electro-oculography (2017 update). Documenta Ophthalmologica. 2017;134(1):1-9.

12. Dasgupta A, Routray A. A new multi-resolution analysis method for electrooculography signals. IEEE Transactions on Neural Systems and Rehabilitation Engineering. 2021.

13. Lopez A, Garcia G, Qaisar SM, Ferrero F, Alvarez JC. EOG compression in polysomnographic recordings based on the Lempel-Ziv-Welch algorithm. Biomedical Signal Processing and Control. 2025;103:107372.

14. Elgendi M, Jonkman M, De Boer F. Frequency bands effects on QRS detection. Biosignals. 2010;2003:2002.

15. Pan J, Tompkins WJ. A real-time QRS detection algorithm. IEEE transactions on biomedical engineering. 1985;(3):230-6.

16. Tompkins WJ. Biomedical digital signal processing: Prentice Hall New Jersey; 1993.

17. Frauscher B, Iranzo A, Gaig C, Gschliesser V, Guaita M, Raffelseder V, et al. Normative EMG values during REM sleep for the diagnosis of REM sleep behavior disorder. Sleep. 2012;35(6):835-47.

18. Sforza E, Johannes M, Claudio B. The PAM-RL ambulatory device for detection of periodic leg movements: a validation study. Sleep medicine. 2005;6(5):407-13.

19. U.S. Food and Drug Administration. 510(k) Premarket Notification: Oxiband (Checkme) O2 Pulse Oximeter, K191088. Silver Spring, MD: U.S. Food and Drug Administration; 2019. Report No.: K191088. Available from: https://www.accessdata.fda.gov/cdrh_docs/pdf19/K191088.pdf.

20. EnsoData, Inc. EnsoSleep Device Profile. Madison, WI: EnsoData, Inc.; 2026. Report No.: REF0007-B-ENG. Available from: https://4433066.fs1.hubspotusercontent-na1.net/hubfs/4433066/ Compliance%20Documents/REF0007-B-ENG_EnsoSleepDeviceProfile.pdf.

21. Seok HS, Shin H. Recommendation for Measuring Digital Volume Pulse in Mobile Application: For Healthy Normal Subject. IEEE Access. 2021;9:69335-45.

22. Viatom Technology. How PPG technology reforms sleep studies and aids in sleep apnea diagnosis [Internet]. 2024 [updated 2025 Apr 30; cited 2026 Jun 19]. Available from: https://www.viatomtech.com/post/how-ppg-technology-reforms-sleep-studies-and-aids-in-sleep-apnea-diagnosis.

23. Roy Y, Banville H, Albuquerque I, Gramfort A, Falk TH, Faubert J. Deep learning-based electroencephalography analysis: a systematic review. Journal of neural engineering. 2019;16(5):051001.

24. Perslev M, Darkner S, Kempfner L, Nikolic M, Jennum PJ, Igel C. U-Sleep: resilient high-frequency sleep staging. NPJ digital medicine. 2021;4(1):72.

25. Retamales G, Gavidia ME, Bausch B, Montanari AN, Husch A, Goncalves J. Towards automatic home-based sleep apnea estimation using deep learning. npj Digital Medicine. 2024;7(1):144.

26. Thapa R, He B, Kjær MR, Moore H, Ganjoo G, Mignot E, et al. SleepFM: multi-modal representation learning for sleep across brain activity, ECG and respiratory signals. arXiv preprint arXiv:240517766. 2024.

27. Fox B, Jiang J, Wickramaratne S, Kovatch P, Suarez-Farinas M, Shah NA, et al. A foundational transformer leveraging full night, multichannel sleep study data accurately classifies sleep stages. Sleep. 2025;48(8). doi: 10.1093/sleep/zsaf061.

28. Park K, Na Y, Choi Y, Ryu H, Shin H-W, Kim H-S. SleepMaMi: A Universal Sleep Foundation Model for Integrating Macro-and Micro-structures. arXiv preprint arXiv:260207628. 2026.
