# Supplementary material for: Polysomnography harmonization schemes: A multi-institutional sleep research resource
Source: PLoS One. 2026 Nov 5;21(11):e0361116. doi: 10.1371/journal.pone.0361116 (PMC13739147; doi:10.1371/journal.pone.0361116)
Supplement: Supplementary file 2 [file pone.0361116.s002.docx]

**Supplementary Table 1. Overview of source datasets used for schema development.** Most datasets include a balanced sex distribution. Meanwhile, SOF consists exclusively of female participants, and MrOS includes only male participants. Both SOF and MrOS enrolled only older adults aged at least 65 years. The CFS dataset focuses on familial predisposition to sleep apnea. It includes a wide age range (6–88 years).

| **Dataset** | **Main study purpose** | **Type** | **Samples (M/F)** | **Age range** |
| --- | --- | --- | --- | --- |
| ABC | Bariatric surgery and CPAP of sleep apnea | I | 49 (28/21) | 26–64 |
| APPLE | Long-term CPAP treatment of sleep apnea | II | 1516 (958/558) | 18–84 |
| CFS | Genetic factors of sleep apnea | I | 735 (329/406) | 6–88 |
| MESA | Gender and ethnicity of sleep disorders | II | 2237 (1039/1198) | 54–95 |
| MrOS | Sleep disorders and risk of falls, fractures, mortality, and vascular disease | II | 8211 (8211/0) | 67–90 |
| SHHS | Sleep and respiratory related cardiovascular disease | II | 5804 (2765/3039) | 40–89 |
| SOF | Dementia, falls, and fractures of osteoporotic | II | 460 (0/460) | 65–89 |
| WSC | Risk factor of sleep disorders and apnea | I | 1123 (608/515) | 37–85 |

ABC: Apnea, Bariatric Surgery, and CPAP Study; APPLE: Apnea Positive Pressure Long-term Efficacy Study; CFS: Cleveland Family Study; MESA: Multi-Ethnic Study of Atherosclerosis; MrOS: Osteoporotic Fractures in Men Study; SHHS: Sleep Heart Health Study; SOF: Study of Osteoporotic Fractures; WSC: Wisconsin Sleep Cohort; M/F: male/female; CPAP: continuous positive airway pressure; Type I: polysomnography records at least 9 channels including electroencephalogram and respiration; Type II: polysomnography is a portable, home-based method with mandatory electroencephalogram.
