# Supplementary material for: Polysomnography harmonization schemes: A multi-institutional sleep research resource
Source: PLoS One. 2026 Nov 5;21(11):e0361116. doi: 10.1371/journal.pone.0361116 (PMC13739147; doi:10.1371/journal.pone.0361116)
Supplement: Supplementary file 3 [file pone.0361116.s003.docx]

**Supplementary Table 2. Schema for biosignal harmonization.** The schema includes the harmonized name, harmonization sampling rate, and original signal labels for each biosignal attribute across datasets. Detailed descriptions of the harmonized biosignal attribute names are provided in Supplementary Table 3. Dashes (–) in table cells indicate that the corresponding biosignal attributes were not available in the examined datasets. Harmonization sampling rates were defined for integrating previously collected heterogeneous PSG datasets and should not be interpreted as acquisition-level sampling recommendations.

| **Type** | **Harmonized name** | **Harmonization sampling rate (Hz)** | **ABC** | **APPLE** | **CFS** | **MESA** | **MrOS** | **SHHS** | **SOF** | **WSC** |
| --- | --- | --- | --- | --- | --- | --- | --- | --- | --- | --- |
| EEG | eeg_f3 | 128 | F3 | – | – | – | – | – | – | – |
|  | eeg_f4 | 128 | F4 | – | – | – | – | – | – | – |
|  | eeg_c3 | 128 | C3 | – | C3 | – | C3 | – | C3 | – |
|  | eeg_c4 | 128 | C4 | – | C4 | – | C4 | – | C4 | – |
|  | eeg_o1 | 128 | O1 | – | – | – | – | – | – | – |
|  | eeg_o2 | 128 | O2 | – | – | – | – | – | – | – |
|  | eeg_m1 | 128 | M1 | – | M1 | – | A1, M1 | – | A1 | – |
|  | eeg_m2 | 128 | M2 | – | M2 | – | A2, M2 | – | A2 | – |
|  | eeg_p3 | 128 | – | – | – | – | – | – | – | – |
|  | eeg_p4 | 128 | – | – | – | – | – | – | – | – |
|  | eeg_f3_m2 | 128 | – | – | – | – | – | – | – | F3_M2 |
|  | eeg_fz_m2 | 128 | – | – | – | – | – | – | – | Fz_M2 |
|  | eeg_c3_m2 | 128 | – | C3_M2 | – | – | – | EEG(sec) | – | C3_M2 |
|  | eeg_c4_m1 | 128 | – | C4_M1 | – | EEG3 | – | EEG | – | – |
|  | eeg_cz_m2 | 128 | – | – | – | – | – | – | – | Cz_M2 |
|  | eeg_o1_m2 | 128 | – | O1_M2 | – | – | – | – | – | O1_M2 |
|  | eeg_o2_m1 | 128 | – | O2_M1 | – | – | – | – | – | – |
|  | eeg_pz_m2 | 128 | – | – | – | – | – | – | – | Pz_M2 |
|  | eeg_fz_cz | 128 | – | – | – | EEG1 | – | – | – | – |
|  | eeg_cz_oz | 128 | – | – | – | EEG2 | – | – | – | – |
| EOG | eog_loc | 128 | E1 | LOC | LOC | EOG-L | LOC, E1 | EOG(L) | LOC | E1 |
|  | eog_roc | 128 | E2 | ROC | ROC | EOG-R | ROC, E2 | EOG(R) | ROC | E2 |
| ECG | ecg_1 | 128 | ECG1 | – | ECG1 | – | ECG L, ECGL | – | ECG1 | – |
|  | ecg_2 | 128 | ECG2 | – | ECG2 | – | ECG R, ECGR | – | ECG2 | – |
|  | ecg_lead2_all | 128 | – | ECG | – | EKG | – | ECG | – | ECG |
| Chin EMG | emg_chin_linked | 256 | – | – | – | EMG | – | – | – | cchin_l, chin |
|  | emg_lchin | 256 | Chin2 | – | EMG2 | – | L Chin, LChin | – | L Chin, EMG/L | – |
|  | emg_rchin | 256 | Chin3 | – | EMG3 | – | R Chin, RChin | – | R Chin, EMG/R | – |
|  | emg_cchin | 256 | Chin1 | – | EMG1 | – | – | – | – | – |
|  | emg_subchin | 256 | – | EMG | – | – | – | EMG | – | – |
| Leg EMG | emg_lleg1 | 256 | LLeg1 | – | – | – | – | – | – | – |
|  | emg_lleg2 | 256 | LLeg2 | – | – | – | – | – | – | – |
|  | emg_rleg1 | 256 | RLeg1 | – | – | – | – | – | – | – |
|  | emg_rleg2 | 256 | RLeg2 | – | – | – | – | – | – | – |
|  | emg_leg | 256 | – | LEG | – | – | – | – | – | lleg_r |
|  | emg_rleg_all | 256 | – | – | – | – | – | – | – | – |
|  | emg_lleg_all | 256 | – | – | – | – | – | – | – | – |
| Limb movement | movement_leg | 64 | – | – | – | LEG | – | – | – | – |
|  | movement_lleg | 64 | – | – | L Leg | – | Leg L | – | Leg/L | – |
|  | movement_rleg | 64 | – | – | R Leg | – | Leg R | – | Leg/R | – |
| Airflow | airflow | 32 | Airflow | – | AIRFLOW | – | Airflow | NEW AIR, AIRFLOW | Airflow | flow |
|  | cannula_flow | 32 | – | – | – | – | Cannula Flow, CannulaFlow | – | Cannula Flow | – |
|  | nasal_pressure | 32 | Nasal Pressure | nasal_pres | NASAL PRES | Flow | - | – | Nasal Pressure | nas_pres |
|  | cpap_flow | 32 | CPAP Flow | – | – | – | - | – | – | – |
|  | cpap_press | 32 | CPAP Press | – | – | – | - | – | – | – |
|  | Thermistor | 32 | – | thermistor | – | Aux_AC, Therm | - | – | – | – |
|  | nasal_flow | 32 | – | – | – | – | - | – | – | nasalflow |
|  | oral_flow | 32 | – | – | – | – | - | – | – | oralflow |
| Respiratory | abdomen | 32 | Abdo | abdomen | ABDO EFFORT | Abdo | Abdominal, ABD | ABDO RES | Abdominal | – |
|  | thorax | 32 | Thor | thorax | THOR EFFORT | Thor | Thoracic, Chest | THOR RES | Thoracic | – |
|  | sum | 32 | Sum | – | SUM | – | – | – | – | – |
|  | snore | 256 | Snore | snore | SNORE | – | – | – | – | snore |
| O2 saturation | oxstat | 1 | Ox Status | – | OX STATUS | OxStatus | STAT | OX stat, OX STAT | STAT | – |
|  | pulse | 1 | Pulse | pulse | PULSE | – | – | – | – | – |
|  | spo2 | 1 | SpO2 | SpO2 | SpO2 | SpO2 | SpO2 | – | – | spO2 |
|  | sao2 | 1 | – | – | SaO2 | – | SaO2, SAO2 | SaO2 | SAO2 | – |
|  | pleth | 128 | Pleth | – | PlethWV | Pleth | – | – | – | – |
| Position | position | 1 | PosSensor | – | POSITION | Pos | Position | POSITION | Position | position |
| Other | hr | 1 | – | – | HRate | HR | HR | H.R., PR | HR | – |
