# Supplementary material for: Polysomnography harmonization schemes: A multi-institutional sleep research resource
Source: PLoS One. 2026 Nov 5;21(11):e0361116. doi: 10.1371/journal.pone.0361116 (PMC13739147; doi:10.1371/journal.pone.0361116)
Supplement: Supplementary file 4 [file pone.0361116.s004.docx]

**Supplementary Table 3. Harmonized biosignal attribute names and descriptions.** For each type, the harmonized name and description of the biosignal attributes are provided. The harmonized names correspond to those listed in Supplementary Table 2.

| **Type** | **Harmonized Name** | **Description** |
| --- | --- | --- |
| EEG | eeg_f3 | Left frontal EEG |
|  | eeg_f4 | Right frontal EEG |
|  | eeg_c3 | Left central EEG |
|  | eeg_c4 | Right central EEG |
|  | eeg_o1 | Left occipital EEG |
|  | eeg_o2 | Right occipital EEG |
|  | eeg_m1 | Left mastoid EEG |
|  | eeg_m2 | Right mastoid EEG |
|  | eeg_p3 | Left parietal EEG |
|  | eeg_p4 | Right parietal EEG |
|  | eeg_f3_m2 | Left frontal to right mastoid EEG |
|  | eeg_fz_m2 | Frontal central to right mastoid EEG |
|  | eeg_c3_m2 | Left central to right mastoid EEG |
|  | eeg_c4_m1 | Right central to left mastoid EEG |
|  | eeg_cz_m2 | Central to right mastoid EEG |
|  | eeg_o1_m2 | Left occipital to right mastoid EEG |
|  | eeg_o2_m1 | Right occipital to left mastoid EEG |
|  | eeg_pz_m2 | Parietal central to right mastoid EEG |
|  | eeg_fz_cz | Frontal to central EEG |
|  | eeg_cz_oz | Occipital to central EEG |
| EOG | eog_loc | Left EOG |
|  | eog_roc | Right EOG |
| ECG | ecg_1 | ECG1 ($-$) |
|  | ecg_2 | ECG2 ($+$) |
|  | ecg_lead2_all | ECG lead II |
| Chin EMG | emg_chin_linked | Linked center & left chin EMG |
|  | emg_lchin | Left chin EMG |
|  | emg_rchin | Right chin EMG |
|  | emg_cchin | Center chin EMG |
|  | emg_subchin | Submentalis EMG |
| Leg EMG | emg_leg | Linked left & right leg EMG |
|  | emg_lleg1 | Left leg EMG ($-$) |
|  | emg_lleg2 | Left leg EMG ($+$) |
|  | emg_lleg_all | Left leg EMG ($-, +$) |
|  | emg_rleg1 | Right leg EMG ($-$) |
|  | emg_rleg2 | Right leg EMG ($+$) |
|  | emg_rleg_all | Right leg EMG ($-, +$) |
| Limb movement | movement_leg | Linked left & right leg movement sensor |
|  | movement_lleg | Left leg movement sensor |
|  | movement_rleg | Right leg movement sensor |
| Airflow | airflow | Airflow |
|  | cannula_flow | Cannula flow |
|  | nasal_pressure | Nasal pressure |
|  | cpap_flow | CPAP flow |
|  | cpap_press | CPAP pressure |
|  | thermistor | Thermistor |
|  | nasal_flow | Nasal airflow |
|  | oral_flow | Oral airflow |
| Respiratory | abdomen | Abdomen |
|  | thorax | Thorax |
|  | sum | Calculated as the mean of abdomen and thorax |
|  | snore | Snore |
| O2 saturation | oxstat | Oximetry status |
|  | pulse | Pulse |
|  | spo2 | SpO_2_ |
|  | sao2 | SaO_2_ |
|  | pleth | Plethysmography |
| Position | position | Position |
| Other | hr | Heart rate |

EEG: electroencephalogram; EOG: electrooculogram; ECG: electrocardiogram; EMG: electromyogram; CPAP: continuous positive airway pressure
