# Supplementary material for: Polysomnography harmonization schemes: A multi-institutional sleep research resource
Source: PLoS One. 2026 Nov 5;21(11):e0361116. doi: 10.1371/journal.pone.0361116 (PMC13739147; doi:10.1371/journal.pone.0361116)
Supplement: Supplementary file 5 [file pone.0361116.s005.docx]

**Supplementary Table 4. Schema for sleep parameter harmonization.** The schema includes the harmonized name and original parameter names for each sleep parameter attribute across datasets. Detailed descriptions of the harmonized sleep parameter attribute names are provided in Supplementary Table 5. Dashes (–) in table cells indicate that the corresponding parameters were not available in the examined datasets. Some harmonized parameter names were predefined based on AASM guidelines and commonly used PSG reporting structures to support extensibility and compatibility with additional PSG datasets, even if corresponding parameters were not present in the currently examined datasets.

| Event type | Harmonized name | ABC | APPLE | CFS | MESA | MrOS | SHHS | SOF | WSC |
| --- | --- | --- | --- | --- | --- | --- | --- | --- | --- |
| Sleep | trt | – | nsrr_ttlprdbd_f1 | timebedp | nsrr_tib_f1 | potimebd | nsrr_ttlprdbd_f1 | nsrr_ttlprdbd_f1 | – |
|  | tst | nsrr_ttldursp_f1 | nsrr_ttldursp_f1 | nsrr_ttldursp_f1 | nsrr_tst_f1 | nsrr_ttldursp_f1 | nsrr_ttldursp_f1 | nsrr_ttldursp_f1 | nsrr_ttldursp_f1 |
|  | lights_out | – | – | – | stloutp5 | – | – | scloutp | – |
|  | lights_on | – | – | – | stlonp5 | – | – | sclonp | – |
|  | sleep_period | – | – | – | – | – | – | – | – |
|  | waso | – | nsrr_ttldurws_f1 | waso | nsrr_waso_f1 | powaso | nsrr_ttldurws_f1 | – | waso |
|  | sleep_onset | – | – | – | stonsetp5 | – | stonsetp | scslpon | – |
|  | sleep_efficiency | – | nsrr_ttleffsp_f1 | slpeffp | nsrr_ttleffsp_f1 | – | slpeffp | nsrr_ttleffsp_f1 | se |
|  | s1 | ttldursp_s1_f1t1 | nsrr_ttldursp_f1*nsrr_pctdursp_s1/100 | minstg1p | timest15 | potmst1 | timest1 | minstg1p | – |
|  | s2 | ttldursp_s2_f1t1 | nsrr_ttldursp_f1*nsrr_pctdursp_s2/100 | minstg2p | timest25 | potmst2 | timest2 | – | – |
|  | s3 | ttldursp_s3_f1t1 | nsrr_ttldursp_f1*nsrr_pctdursp_s3/100 | minstg34p | timest345 | potmst34 | timest34 | – | – |
|  | r | ttldursp_sr_f1t1 | nsrr_ttldursp_f1*nsrr_pctdursp_sr/100 | minremp | timerem5 | potmrem | – | minremp | – |
|  | w | – | – | – | – | – | – | – | – |
|  | sleep_latency | – | nsrr_ttllatsp_f1 | slplatp | nsrr_ttllatsp_f1 | – | nsrr_ttllatsp_f1 | nsrr_ttllatsp_f1 | sleep_latency |
|  | sleep_latency_to_n1 | – | – | – | – | – | – | – | – |
|  | sleep_latency_to_n2 | – | – | – | – | – | – | – | – |
|  | sleep_latency_to_n3 | – | – | – | – | – | – | – | – |
|  | sleep_r_latency_from_sleep_onset | – | nsrr_ttlprdsp_s1sr | remlaip | nsrr_ttlprdsp_s1sr | – | nsrr_ttlprdsp_s1sr | nsrr_ttlprdsp_s1sr | rem_latency |
|  | s1_tst | pctdursp_s1_f1t1 | nsrr_pctdursp_s1 | minstg1p/nsrr_ttldursp_f1 | nsrr_pctdursp_s1 | potmst1p | nsrr_pctdursp_s1 | nsrr_pctdursp_s1 | pcttststagen1 |
|  | s2_tst | pctdursp_s2_f1t1 | nsrr_pctdursp_s2 | minstg2p/nsrr_ttldursp_f1 | nsrr_pctdursp_s2 | potmst2p | nsrr_pctdursp_s2 | nsrr_pctdursp_s2 | pcttststagen2 |
|  | s3_tst | pctdursp_s3_f1t1 | nsrr_pctdursp_s3 | minstg34p/nsrr_ttldursp_f1 | nsrr_pctdursp_s3 | potms34p | nsrr_pctdursp_s3 | nsrr_pctdursp_s3 | pcttststage34 |
|  | sr_tst | pctdursp_sr_f1t1 | nsrr_pctdursp_sr | minremp/nsrr_ttldursp_f1 | nsrr_pctdursp_sr | potmremp | nsrr_pctdursp_sr | nsrr_pctdursp_sr | pcttstrem |
|  | s1_sleep_period | – | – | – | – | – | – | – | – |
|  | s2_sleep_period | – | – | – | – | – | – | – | – |
|  | s3_sleep_period | – | – | – | – | – | – | – | – |
|  | sr_sleep_period | – | – | – | – | – | – | – | – |
|  | w_sleep_period | – | – | – | – | – | – | – | – |
|  | sleep_stage_5 | from annotation | from annotation | from annotation | from annotation | from annotation | from annotation | from annotation |  |
|  | sleep_stage_3 | from annotation | from annotation | from annotation | from annotation | from annotation | from annotation | from annotation |  |
| Arousal | apnea_arousals_num | – | – | – | – | – | – | – | – |
|  | apnea_arousals_idx | – | – | – | – | – | – | – | – |
|  | lm_arousals_num | – | – | – | – | – | – | lmaslp | – |
|  | lm_arousals_idx | – | – | – | – | – | – | – | – |
|  | desaturation_num | – | – | – | – | – | – | – | – |
|  | desaturation_idx | – | – | – | – | – | – | – | – |
|  | respiratory_arousals_num | – | – | – | – | – | – | – | – |
|  | respiratory_arousals_idx | – | – | – | – | – | – | – | – |
|  | user_defined_arousals_num | – | – | – | – | – | – | – | – |
|  | user_defined_arousals_idx | – | – | – | – | – | – | – | – |
|  | spontaneous_arousals_num | – | – | – | – | – | – | – | – |
|  | spontaneous_arousals_idx | – | – | – | – | – | – | – | – |
|  | hypopnea_arousals_num | – | – | – | – | – | – | – | – |
|  | hypopnea_arousals_idx | – | – | – | – | – | – | – | – |
|  | plm_arousals_num | – | – | – | plmaslp5 | – | – | plmaslp | – |
|  | plm_arousals_idx | – | – | – | – | – | – | – | – |
|  | snore_arousals_num | – | – | – | casndur5 | – | – | – | – |
|  | snore_arousals_idx | – | – | – | – | – | – | – | – |
|  | total_arousals_num | – | arousalrem+arousalnrem | – | – | – | – | – | – |
|  | total_arousals_idx | – | nsrr_phrnumar_f1 | nsrr_phrnumar_f1 | nsrr_phrnumar_f1 | nsrr_phrnumar_f1 | nsrr_phrnumar_f1 | nsrr_phrnumar_f1 | – |
| Respiratory | ahi | nsrr_ahi_hp3r_aasm15 | nsrr_ahi_chicago1999 | nsrr_ahi_hp3r_aasm15 | nsrr_ahi_hp3r_aasm15 | nsrr_ahi_hp3r_aasm15 | nsrr_ahi_hp3r_aasm15 | nsrr_ahi_hp3r_aasm15 | – |
|  | total_apnea_num | – | – | – | – | – | – | – | – |
|  | total_apnea_idx | – | – | – | – | – | – | – | – |
|  | oa_num | – | respnumoapsg | oai0p*nsrr_ttldursp_f1/60 | – | – | – | – | – |
|  | oa_idx | oai_oa0u_f1t1 | oaindextstpsg | oai0p | oai0p5 | pooai0p | oai0p | oai0p | – |
|  | ca_num | – | respnumcapsg | cai0p*nsrr_ttldursp_f1/60 | – | – | – | – | – |
|  | ca_idx | cai_ca0u_f1t1 | caindextstpsg | cai0p | cai0p5 | pocai0p | cai0p | cai0p | – |
|  | ma_num | – | respnummapsg | – | – | – | – | – | – |
|  | ma_idx | – | maindextstpsg | – | – | – | – | – | – |
|  | oh_num | – | – | – | – | – | – | – | – |
|  | oh_idx | – | – | – | – | – | – | – | – |
|  | ch_num | – | – | – | – | – | – | – | – |
|  | ch_idx | – | – | – | – | – | – | – | – |
|  | total_hypopnea_num | – | respnumhyppsg | – | – | – | – | – | – |
|  | total_hypopnea_idx | hi_hp3x0u_f1t1 | hypindextstpsg | – | – | – | – | – | – |
|  | oahi_num | – | – | – | – | – | – | – | – |
|  | oahi_idx | oahi_oa0uhp3x3r_f1t1 | – | ahi_o0h3a | oahi3pa5 | poordi3 | ahi_o0h3a | ahi_o0h3a | – |
|  | cahi_num | – | – | – | – | – | – | – | – |
|  | cahi_idx | cahi_ca0uhp3x3r_f1t1 | – | ahi_c0h3a | – | pocahi3 | ahi_c0h3a | ahi_c0h3a | – |
|  | rdi_idx | – | – | – | – | – | – | – | – |
|  | rera_num | – | – | – | – | – | – | – | – |
|  | rera_idx | – | – | – | – | – | – | – | – |
|  | odi_idx | – | desatindextot | – | – | – | – | – | – |
|  | snore_time | – | – | – | – | – | – | – | – |
|  | snore_pct | – | – | – | – | – | – | – | – |
|  | hypoventilation_num | – | – | – | – | – | – | – | – |
|  | hypoventilation_idx | – | – | – | – | – | – | – | – |
|  | cheyne_stokes_num | – | – | – | – | – | – | – | – |
|  | cheyne_stokes_tst | – | – | – | – | – | – | – | – |
|  | rmi_num | – | – | – | – | – | – | – | – |
|  | rmi_idx | – | – | – | – | – | – | – | – |
|  | periodic_breathing_num | – | – | – | period5 | – | – | – | – |
|  | periodic_breathing_idx | – | – | – | – | – | – | – | – |
| Body  movement | total_lm_num | – | – | lmar | – | – | – | lmtot | – |
|  | total_lm_idx | – | – | – | – | – | – | – | – |
|  | total_plm_num | – | – | – | noplm5 | poplmslp | – | – | – |
|  | total_plm_idx | – | – | – | – | poavgplm | – | – | – |
|  | supine_num | – | – | – | – | – | – | – | – |
|  | supine_idx | – | – | – | – | – | – | – | – |
|  | prone_num | – | – | – | – | – | – | – | – |
|  | prone_idx | – | – | – | – | – | – | – | – |
|  | right_num | – | – | – | – | – | – | – | – |
|  | right_idx | – | – | – | – | – | – | – | – |
|  | upright_num | – | – | – | – | – | – | – | – |
|  | upright_idx | – | – | – | – | – | – | – | – |
|  | left_num | – | – | – | – | – | – | – | – |
|  | left_idx | – | – | – | – | – | – | – | – |
|  | unknown_num | – | – | – | – | – | – | – | – |
|  | unknown_idx | – | – | – | – | – | – | – | – |
|  | plm_lm_num | – | plmcriteriaindex | noplm | – | – | – | noplm | – |
|  | plm_lm_idx | – | – | – | – | – | – | – | – |
|  | plm_rrlm_num | – | – | – | – | – | – | – | – |
|  | plm_rrlm_idx | – | – | – | – | – | – | – | – |
|  | alma_num | – | – | – | – | – | – | – | – |
|  | alma_idx | – | – | – | – | – | – | – | – |
|  | hft_num | – | – | – | – | – | – | – | – |
|  | hft_idx | – | – | – | – | – | – | – | – |
|  | active_move_num | – | – | – | – | – | – | – | – |
|  | active_move_idx | – | – | – | – | – | – | – | – |
| Cardiac | average_heart_rate_during_sleep | avglvlhr_f1t1 | – | – | bpmavg5 | – | – | – | – |
|  | average_heart_rate_during_recording | – | – | Bpm  avg | – | – | – | – | – |
|  | highest_heart_rate_during_sleep | – | – | – | bpmmax5 | pobpmmax | – | – | – |
|  | highest_heart_rate_during_recording | – | – | bpmmax | – | – | – | – | – |
|  | lowest_heart_rate_during_sleep | – | – | – | bpmmin5 | pobpmmin | – | – | – |
|  | lowest_heart_rate_during_recording | – | – | bpmmin | – | – | – | – | – |
|  | heartrate_increase_num | – | – | – | – | – | – | – | – |
|  | asystole_num | – | – | – | – | – | – | – | – |
|  | sinus_tachycardia_num | – | – | – | – | – | – | – | – |
|  | narrow_complex_tachycardia_num | – | – | – | – | – | – | – | – |
|  | wide_complex_tachycardia_num | – | – | – | – | – | – | – | – |
|  | bradycardia_num | – | – | nobrh | – | ponbrslp | – | – | – |
|  | atrial_fibrillation_num | – | – | – | – | – | – | – | – |
|  | other_arrhythmia_num | – | – | – | – | – | – | – | – |
