# Supplementary material for: Polysomnography harmonization schemes: A multi-institutional sleep research resource
Source: PLoS One. 2026 Nov 5;21(11):e0361116. doi: 10.1371/journal.pone.0361116 (PMC13739147; doi:10.1371/journal.pone.0361116)
Supplement: Supplementary file 6 [file pone.0361116.s006.docx]

**Supplementary Table 5. Harmonized sleep parameter attribute names and descriptions.** For each event type, the harmonized name and description of the sleep parameter attributes are provided. The harmonized names correspond to those listed in Supplementary Table 4.

| **Event Type** | **Harmonized name** | **Description** |
| --- | --- | --- |
| Sleep | trt | TRT |
|  | tst | TST |
|  | lights_out | Lights out clock time |
|  | lights_on | Lights on clock time |
|  | sleep_period | Sleep period (= TRT - Sleep onset) |
|  | waso | WASO |
|  | sleep_onset | Sleep onset |
|  | sleep_efficiency | Sleep efficiency [%] (= (TST / TRT) × 100) |
|  | s1 | Total N1 stage time |
|  | s2 | Total N2 stage time |
|  | s3 | Total N3 stage time |
|  | r | Total REM stage time |
|  | w | Total wake stage time |
|  | sleep_latency | Interval between lights-out/in-bed time and sleep onset |
|  | sleep_latency_to_n1 | Sleep latency to N1 |
|  | sleep_latency_to_n2 | Sleep latency to N2 |
|  | sleep_latency_to_n3 | Sleep latency to N3 (= SWS) |
|  | sleep_r_latency_from_sleep_onset | Sleep R latency from Sleep onset |
|  | s1_tst | Ratio of Total N1 stage time to TST |
|  | s2_tst | Ratio of Total N2 stage time to TST |
|  | s3_tst | Ratio of Total N3 stage time to TST |
|  | sr_tst | Ratio of Total REM stage time to TST |
|  | s1_sleep_period | Ratio of Total N1 stage time to Sleep period |
|  | s2_sleep_period | Ratio of Total N2 stage time to Sleep period |
|  | s3_sleep_period | Ratio of Total N3 stage time to Sleep period |
|  | sr_sleep_period | Ratio of Total REM stage time to Sleep period |
|  | w_sleep_period | Ratio of Total WAKE stage time to Sleep period |
|  | sleep_stage_5 | Five sleep stage classification based on 30 s epochs: 0 = Wake, 1 = N1, 2 = N2, 3 = N3, 4 = REM |
|  | sleep_stage_3 | Three sleep stage classification based on 30 s epochs: 0 = Wake, 1 = NREM, 2 = REM |
| Arousal | apnea_arousals_num | Number of apnea arousals |
|  | apnea_arousals_idx | Number of apnea arousals per hour of sleep |
|  | lm_arousals_num | Number of leg movement arousals |
|  | lm_arousals_idx | Number of leg movement arousals per hour of sleep |
|  | desaturation_num | Number of desaturation arousals |
|  | desaturation_idx | Number of desaturation arousals per hour of sleep |
|  | respiratory_arousals_num | Number of respiratory arousals |
|  | respiratory_arousals_idx | Number of respiratory arousals per hour of sleep |
|  | user_defined_arousals_num | Number of unspecified arousals |
|  | user_defined_arousals_idx | Number of unspecified arousals per hour of sleep |
|  | spontaneous_arousals_num | Number of spontaneous arousals |
|  | spontaneous_arousals_idx | Number of spontaneous arousals per hour of sleep |
|  | hypopnea_arousals_num | Number of hypopnea arousals |
|  | hypopnea_arousals_idx | Number of hypopnea arousals per hour of sleep |
|  | plm_arousals_num | Number of PLM arousals |
|  | plm_arousals_idx | Number of PLM arousals per hour of sleep |
|  | snore_arousals_num | Number of snore arousals |
|  | snore_arousals_idx | Number of snore arousals per hour of sleep |
|  | total_arousals_num | Number of total arousals |
|  | total_arousals_idx | Number of total arousals per hour of sleep |
| Respiratory | ahi | AHI |
|  | total_apnea_num | Number of total apneas |
|  | total_apnea_idx | AI |
|  | oa_num | Number of obstructive apneas |
|  | oa_idx | OAI |
|  | ca_num | Number of central apneas |
|  | ca_idx | CAI |
|  | ma_num | Number of mixed apneas |
|  | ma_idx | MAI |
|  | oh_num | Number of obstructive hypopneas |
|  | oh_idx | OHI |
|  | ch_num | Number of central hypopneas |
|  | ch_idx | CHI |
|  | total_hypopnea_num | Number of total hypopneas |
|  | total_hypopnea_idx | HI |
|  | oahi_num | Number of obstructive apnea hypopnea |
|  | oahi_idx | OAHI |
|  | cahi_num | Number of central apnea hypopneas |
|  | cahi_idx | CAHI |
|  | rdi_idx | RDI |
|  | rera_num | Number of RERAs |
|  | rera_idx | Number of RERAs per hour of sleep |
|  | odi_idx | ODI |
|  | snore_time | Total snore time |
|  | snore_pct | Percentage of total snore time |
|  | hypoventilation_num | Number of hypoventilations |
|  | hypoventilation_idx | Number of hypoventilations per hour of sleep |
|  | cheyne_stokes_num | Number of cheyne-stokes |
|  | cheyne_stokes_tst | Number of cheyne-stokes per hour of sleep |
|  | rmi_num | Number of respiratory movement |
|  | rmi_idx | RMI |
|  | periodic_breathing_num | Number of periodic breathings |
|  | periodic_breathing_idx | Number of periodic breathings per hour of sleep |
| Bodymovement | total_lm_num | Number of total movements without arousal |
|  | total_lm_idx | Number of total movements per hour of sleep |
|  | total_plm_num | Number of total periodic limb movements of sleep |
|  | total_plm_idx | Number of PLMs per hour of sleep |
|  | supine_num | Number of supine movements |
|  | supine_idx | Number of supine movements per hour of sleep |
|  | prone_num | Number of prone movements |
|  | prone_idx | Number of prone movements per hour of sleep |
|  | right_num | Number of right movements |
|  | right_idx | Number of right movements per hour of sleep |
|  | upright_num | Number of upright movements |
|  | upright_idx | Number of upright movements per hour of sleep |
|  | left_num | Number of unspecified movements |
|  | left_idx | Number of unspecified movements per hour of sleep |
|  | unknown_num | Number of left movements |
|  | unknown_idx | Number of left movements per hour of sleep |
|  | plm_lm_num | Number of periodic limb leg movements |
|  | plm_lm_idx | Number of periodic limb leg movements per hour of sleep |
|  | plm_rrlm_num | Number of respiratory event-related limb movements |
|  | plm_rrlm_idx | Number of respiratory event-related limb movements per hour of sleep |
|  | alma_num | Number of alternating leg muscle activations |
|  | alma_idx | Number of alternating leg muscle activations per hour of sleep |
|  | hft_num | Number of hypnagogic foot tremors |
|  | hft_idx | Number of hypnagogic foot tremors per hour of sleep |
|  | active_move_num | Number of active movements |
|  | active_move_idx | Number of active movements per hour of sleep |
| Cardiac | average_heart_rate_during_sleep | average heart rate during total sleep time |
|  | average_heart_rate_during_recording | average heart rate during total recording time |
|  | highest_heart_rate_during_sleep | max heart rate during total sleep time |
|  | highest_heart_rate_during_recording | max heart rate during total recording time |
|  | lowest_heart_rate_during_sleep | min heart rate during total sleep time |
|  | lowest_heart_rate_during_recording | min heart rate during total recording time |
|  | heartrate_increase_num | Number of rising heart rate |
|  | asystole_num | Number of asystole |
|  | sinus_tachycardia_num | Number of sinus tachycardia |
|  | narrow_complex_tachycardia_num | Number of narrow complex tachycardia |
|  | wide_complex_tachycardia_num | Number of wide complex tachycardia |
|  | bradycardia_num | Number of bradycardia |
|  | atrial_fibrillation_num | Number of atrial fibrillation |
|  | other_arrhythmia_num | Number of other arrhythmia |

TRT: total recording time; TST: total sleep time; WASO: wake after sleep onset; SWS: slow wave sleep; PLM: periodic leg movements; AHI: apnea-hypopnea index; AI: apnea index; OAI: obstructive apnea index; CAI: central apnea index; MAI: mixed apnea index; OHI: obstructive hypopnea index; CHI: central hypopnea index; HI: hypopnea index; OAHI: obstructive apnea hypopnea index; CAHI: central apnea hypopnea index; RERAs: respiratory effort-related arousal; RDI: respiratory disturbance index; ODI: oxygen desaturation index, number of desaturation events (≥ 3% or ≥ 4%) per hour of sleep; RMI: respiratory movement index. Index refers to the number of events per hour of total sleep time.
